# Predictive Factors for Natural Pregnancy after Microsurgical Reconstruction in Patients with Primary Epididymal Obstructive Azoospermia

**DOI:** 10.1155/2014/873527

**Published:** 2014-06-01

**Authors:** Mihai Harza, Sebastian Voinea, Gener Ismail, Cristian Gagiu, Catalin Baston, Adrian Preda, Ioan Manea, Tiberiu Priporeanu, Ioanel Sinescu

**Affiliations:** ^1^Center for Uronephrology and Renal Transplantation, Fundeni Clinical Institute, 258 Fundeni Street, District 2, 022328 Bucharest, Romania; ^2^Center of Internal Medicine-Nephrology, Fundeni Clinical Institute, 022328 Bucharest, Romania

## Abstract

Primary epididymal obstructive azoospermia (OA) is the most prevalent form of OA in nonvasectomized patients and has been less studied. We aim to assess the results with microsurgical vasoepididymostomy used in the treatment of men diagnosed with primary epididymal obstructive azoospermia and to identify the factors associated with natural pregnancy occurring after microsurgical reconstruction. This prospective study included consecutive patients with epididymal OA who underwent microsurgical reconstruction in our center. Clinical and biological data were obtained every three months during follow-up. Occurrence of natural pregnancy was the primary study outcome. In total, 36 patients underwent microsurgical reconstruction. The mean age was 34 ± 4.5 years (range 24–46 years). Median follow-up time was 15 [IQR 12–21] months. The total patency rate was 77.7% (*n* = 28). During follow-up, 8 (22.2%) natural pregnancies occurred. The overall live birth rate was 100%. Low FSH levels (HR: 0.22; 95% CI: 0.052–0.88; *P* = 0.032) and higher total motile sperm count (TMSC) (HR: 1.001; 95% CI 1–1.001; *P* = 0.012) were associated with a higher rate of natural pregnancy. Our data suggest that microsurgical vasoepididymostomy is an effective therapy of primary epididymal OA. Baseline lower FSH and higher TMSC were independent predictors for natural pregnancy occurrence.

## 1. Introduction

Obstructive azoospermia (OA) is present in 10–20% of infertile men and is fundamentally characterized by normal testicular volume and reproductive hormones indicating normal spermatogenesis [1]. While postvasectomy OA has been extensively reported by many authors, primary OA (unrelated to a prior vasectomy) is present in about 3%–6% of all infertile men and most frequently is produced by epididymal obstruction [2]. Primary OA (mainly after sexually transmitted diseases and rarely after iatrogenic lesion of the male genital tract) or congenital OA (bilateral absence of the vas deferens and idiopathic epididymal obstruction) may be acquired. Both microsurgical reconstruction of the spermatic tract and sperm retrieval followed by intracytoplasmic sperm injection (ICSI) could be beneficial for such patients. Nowadays, urologist specialized in infertility should be able to accurately predict which patients have normal spermatogenesis and/or are suitable for surgical reconstruction. In the evaluation algorithm of patients with suspected OA, different noninvasive and invasive tests can be approached. Normal serum concentration of follicle-stimulating hormone (FSH), a traditional marker for spermatogenesis, is the first step that differentiates primitive OA from non-OA [3–6]. Also, seminal anti-Müllerian hormone and plasma antisemen antibodies are other potential noninvasive predictive markers for the presence of spermatogenesis in patients with suspected OA [7–9]. Percutaneous epididymal sperm aspirationhas a high rate of sperm retrieval for ICSI regardless of the etiology of OA [10] but with the risk of subsequent scaring that might end with a second obstruction located in the epididymal head nonfeasible for microsurgical reconstruction [11]. Testicular fine needle aspiration (TFNA) has been traditionally used to retrieve sperm in non-OA. Recently it was established that a primary azoospermic male with proven normal spermatogenesis by TFNA and a normal FSH level should be diagnosed as OA and explored for reconstruction [12]. Testicular sperm extraction (TESE) is the best invasive procedure to predict which cases are suitable for microsurgical reconstruction but still cannot predict its results [13].

The aim of this study was to assess our results obtained by microsurgical vasoepididymostomy (VES) in the treatment of men diagnosed with primary epididymal OA and to identify the factors associated with natural pregnancy occurring after microsurgical reconstruction.

## 2. Materials and Methods

### 2.1. Study Design

This was a prospective single-center study.

The patients were included in the study, if they had OA secondary to epididymal obstruction and fulfilled all the inclusion criteria (*vide infra*). Data from the admitted patients were obtained at baseline and every three months.

The study end point was the occurrence of a natural pregnancy confirmed by appropriate diagnostic tools. In patients without a natural pregnancy, the follow-up was at least 12 months.

The local ethics committee approved the study protocol, and all the patients gave an informed consent at admission.

### 2.2. Patients

All consecutive patients with OA secondary to epididymal obstruction admitted to our department between September 2008 and October 2012 in whom microsurgical reconstruction was achievable were considered for inclusion (*n* = 65).

The diagnosis criteria of OA secondary to epididymal obstruction were azoospermia diagnosed on at least 2 semen analyses after centrifugation, vas deferens clinically present, seminal volume ≥1 mL, semen pH ≥7, seminal fructose present, normal serum level of follicle stimulating hormone (FSH), luteinizing hormone (LH) and testosterone, at least one testicle with a volume ≥10 cc, and the width of the epididymis caput ≥5 mm, as measured by ultrasound [14].

Microsurgical reconstruction was considered feasible in all patients who fulfilled the following criteria: (1) at least one viable spermatozoon in the aspirated fluid by microsurgical epididymal sperm aspiration (MESA), (2) a dilated epididymal tubule located cranially to the level of viable spermatozoa aspirated by MESA, and (3) a patent vas deferens. No exploration of the vas deferens was performed if criteria 1 and 2 were not achieved. A dilated/not dilated tubule was defined as a tubule large/not large enough to allow placement of the needles used in reconstruction.

Patients were excluded if they had a history of vasectomy, attempted microsurgical reconstruction and pre/intraoperative diagnostic of bilateral congenital absence of vas deferens, infertile female partner, and lack of desire to have children.

The patency was defined as a concentration of more than 0.1 × 10^6^ sperms/mL.

**Figure 1:**
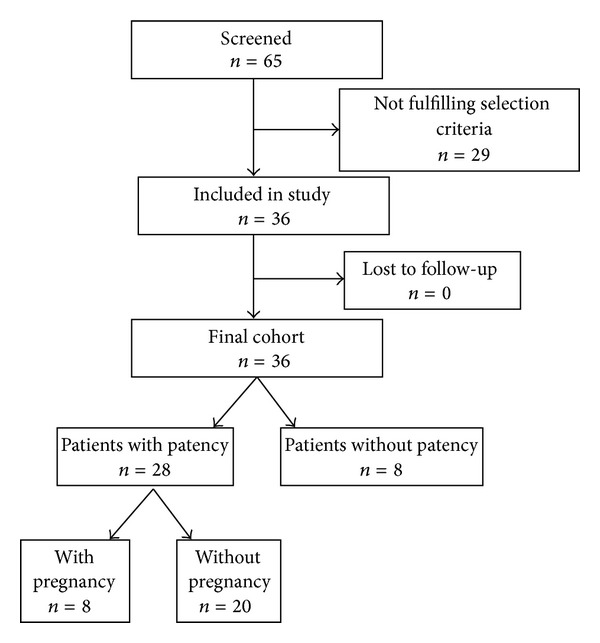
Patient selection.

The final study population included 36 patients with primary epididymal OA who underwent microsurgical reconstruction (Figure 1).

### 2.3. Parameters

The patient's examined characteristics included age, age of female partners, smoking status, testicular volume, thickness of the right and left epididymis, total motile sperm count (TMSC), and serum reproductive hormones (FSH, LH, testosterone). Other parameters of interest were prior fertility status (natural pregnancy in the past history) and medical history of urological infections.

All semen specimens were collected by masturbation at the Clinical Andrology Laboratory after a period of 3–5 days of abstinence.

The patients who underwent microsurgical reconstruction were advised to wait until the end of the postoperative follow-up period before taking any other treatment option for infertility.

#### 2.3.1. Surgery

A microsurgical transversal end-to-side double arm single tubule VES was performed as was previously described by Marmar [15]. Surgery was performed under general anesthesia. After creating a longitudinal raphe scrotal incision, the testicle was delivered. The vaginalis tunica was opened and reversed. The tail of the epididymis was identified and a microscope (optical magnification ×12–×30) was brought into the operatory field. A single epididymal tubule was exposed as caudally as possible. The tubule was opened, MESA was performed, and the epididymal fluid was examined in the operating room for viable spermatozoa using light microscopy. If they were present, the fluid was sent for cryopreservation and VES was attempted if the epididymal tubule located 5 mm cranially was dilated. The vas deferens and its pedicle were transected transversally as close as possible to the testicle, but in its straight part, and dissected cranially 3-4 cm to obtain a tension-free anastomosis with the epididymis. The deferent duct was catheterized with a 24-gauge angiocatheter and methylene blue was injected. The vas deferens was considered if the injection was easy and the urine turned green in the collection bag. In case of patent vas deferens, a single epididymal tubule was dissected 5 mm cranially to the level where the viable sperms were identified. Two parallel double needles (6.5 mm) 10.0, 30 cm long sutures (Ethicon) were placed in the selected epididymal tubule, oriented transversally. The tubule was incised between the two needles. The double-arm needles were placed in-to-out through the vassal lumen finishing the anastomosis. If the motile spermatozoa were not present, more proximal MESA was performed until motile spermatozoa were identified. If viable spermatozoa were not identified along the entire epididymis, a multisite testicular biopsy (TESE) was performed and sent for sperm cryopreservation. No exploration of the rete testis was performed. The same procedure was performed on the opposite side if the testis was present.

#### 2.3.2. Statistical Analysis

Continuous variables are presented as mean or median and 95% confidence interval, according to their distribution, and categorical variables as percentages. Group comparisons were performed with Student's *t*-test, *χ* ^2^ test, and Mann-Whitney *U* test, as appropriate.

Receiver operator characteristic (ROC) curve analysis was performed to evaluate the utility and to identify cut-off values for parameters significantly correlated with the natural pregnancy occurrence.

A multivariable Cox regression analysis was performed in order to determine the factors associated with the natural pregnancy occurrence. Results were expressed as hazard ratios (Exp(B)) with 95% CIs. Hazard ratios are expressed per natural logarithm unit of TMSC, per unit of age, FSH, LH, and testosterone, and dichotomized for prior fertility status and medical history of urological infections. A *P* ≤ 0.05 was considered statistically significant. Analyse-it (Analyse-it Software, Ltd., Leeds, UK) and SPSS (SPSS Inc., Chicago, IL, USA) software were used to analyze the data.

## 3. Results

### 3.1. Clinical Baseline Characteristics

Thirty-six patients with primary OA secondary to epididymal obstruction who underwent microsurgical reconstruction were included. Baseline characteristics of the study population are displayed in Table 1. At the time of study inclusion, the median age of patients was 34 [IQR 31–37] years and the median age of female partners was 32 [IQR 29–36] years. Median follow-up time was 15 [IQR 12–21] months.

The total patency rate in the study group was 77.7% (*n* = 28). In the group of patients with patent anastomosis, 4 (14.3%) patients had biological father status and 15 (53.6%) patients had medical history of infection of the male genital tract.

### 3.2. Factors Predicting Natural Pregnancy Occurrence

During the follow-up period, no patient was lost and in 8 (28.6%), out of 28 subjects with patent anastomosis, natural pregnancies occurred with an overall live birth rate of 100%. In univariate analysis, as compared to those without natural pregnancy, the patients with natural pregnancy had significantly higher level of the total motile sperm count (TMSC) (*P* = 0.0001). Also, patients with natural pregnancy during the follow-up period had significantly lower FSH levels when compared with patients with no natural pregnancy (*P* = 0.02) (Table 1). In contrast, baseline LH and testosterone levels were not associated with natural pregnancy (*P* = 0.75, resp., *P* = 0.70). Also, testicular volume and thickness of the right and left epididymis were not significantly associated with natural pregnancy (Table 1).

The median age of female partners with and without natural spontaneous pregnancy was 30 and 31.5 years (*P* = 0.41), respectively.

History of an infection of the male genital tract showed an inverse relationship with pregnancy occurrence, although not significant (*P* = 0.38). Also, a positive prior fertility status showed a direct relationship with pregnancy occurrence, although not significant (*P* = 0.56).

Next we performed a multivariate analysis, including age, age of female partners, FSH, LH, testosterone, TMSC levels, prior fertility status, and medical history of urological infections at the time of study start. The results of multivariable Cox regression analysis are shown in Table 2. According to multivariate analysis, two variables were independently related to increased rates of natural pregnancy occurrence after microsurgical reconstruction: higher TMSC and low FSH level at the time of study start. An increase of TMSC by 1 natural logarithm unit was associated with a 1.001-fold increase of spontaneous pregnancy rates (95% CI: 1–1.001). An increase of FSH by 1 UI/L was associated with a 0.22-fold increase of spontaneous pregnancy rates (95% CI: 0.052–0.88) (Table 2).

**Figure 2:**
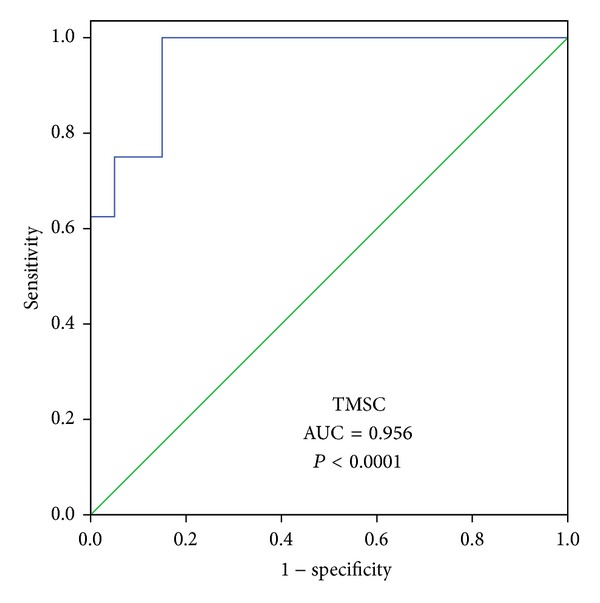
TMSC diagnostic utility for natural pregnancy occurrence (ROC analysis).

**Figure 3:**
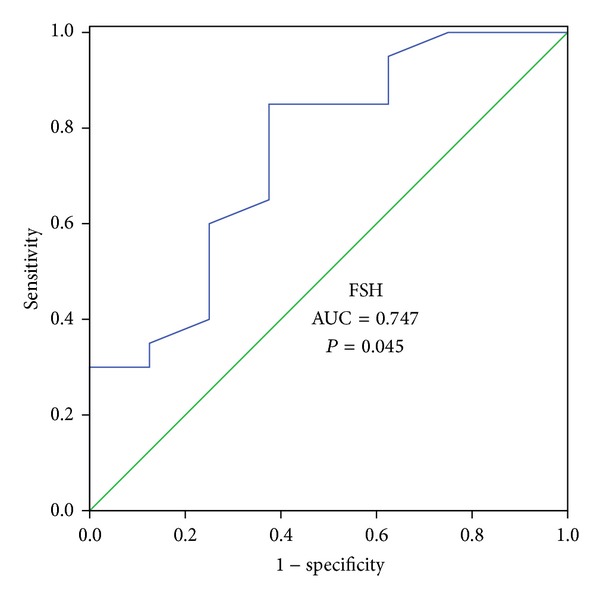
FSH diagnostic utility for natural pregnancy occurrence (ROC analysis).

ROC curve analysis was performed to identify the cut-off values for TMSC and FSH levels which predict a natural pregnancy occurrence after microsurgical reconstruction. Thus, a TMSC of 37.30 × 10^6^ has a sensitivity of 87.5% and a specificity of 85% to predict a spontaneous pregnancy occurrence after microsurgical reconstruction (Figure 2). Also, a FSH level of 2.6 UI/L has a sensitivity of 85% and a specificity of 62.5% to predict a spontaneous pregnancy occurrence after microsurgical reconstruction (Figure 3).

## 4. Discussions

Sperm retrieval and ICSI have dramatically improved the chances for patients diagnosed with azoospermia to obtain a pregnancy [16]. Although ICSI can be used in patients with azoospermia of any etiology, it has already been proven that microsurgical reconstruction of the genital tract in patients with a history of vasectomy is the preferred approach, with reported patency rates of 67%–85% [17, 18] and spontaneous pregnancy of 27%–49% [18, 19]. Although the results of the reconstructions are essentially the same for patients with primary epididymal obstruction, many gynecologists and endocrinologists are not aware of this data. Kim et al. [20] reported the results of 43 end-to-side VES in patients with primary epididymal obstruction unrelated to a prior vasectomy. After a mean follow-up period of 42 months, the patency and pregnancy rates were 81% and 37%, respectively. Paick et al. [21] reported the results of 61 patients with primitive obstructive azoospermia who underwent microsurgical single tubule VES. After a minimum of 24 months follow-up period the overall patency and natural birth rates were 68.9% and 21.3%, respectively. In our study, after a median period of follow-up of 15 months, the total patency and natural birth rates were 77.7% and 22.2%, respectively. The time of follow-up in our study was shorter than that in the cited studies which might affect our pregnancy rates. As it was previously reported, microsurgical reconstruction is not the first option for couples where the male partner is diagnosed with OA and the female partner is >39 years old and has a low ovarian reserve [22]. In our study, the mean age of female partners in the group of patients with patent anastomosis and no natural pregnancy was 32.5 years.

Natural pregnancy in the past history of a man with OA is a strong evidence of normal spermatogenesis before obstruction occurrence. In our study, a positive prior fertility status was found in 4 patients. Our results show a direct relationship between positive prior fertility status and pregnancy occurrence, although not significant (*P* = 0.56), very probable because of a low number of patients.

After microsurgical reconstruction for azoospermia, if the controls 1–3 months postoperatively show some sperm, the couple is inclined to perform ICSI using fresh ejaculated sperm. Predicting natural pregnancy after reconstruction is understandable because it helps counseling the patients after reconstruction. ICSI not only is costly, but also is invasive for women and carries some risks for offspring. So it would be advisable to know when to choose ICSI and when to wait for a natural pregnancy. Our study shows that, in patients with primitive epididymal OA treated with reconstruction, FSH level is a strong predictive factor for obtaining a natural pregnancy. A FSH of 2.6 UI/L has a sensitivity of 85% and a specificity of 62.5% to predict a spontaneous pregnancy occurrence after microsurgical reconstruction. To our knowledge, there are no published studies which report the role of FSH in predicting a natural pregnancy after reconstruction. The potential mechanism of FSH level in impacting spontaneous pregnancy would be the fact that a low FSH level reflects a good spermatogenesis before and after obstruction occurrence. TMSC has been recognized as an important factor which predicts a pregnancy occurrence. Some studies attempted to find a correlation between the sperm parameter and natural pregnancy in a group of primitive epididymal OA [20, 21]. Kim et al. found higher sperm density and motility in postoperative semen analyses in patients who obtained natural pregnancy versus no natural pregnancy, but the difference was not significant. Paick et al. reported the results of reconstruction by VES in 61 patients with primitive epididymal OA. Out of 13 live deliveries by natural conception, 9 occurred in a group of patients with postoperative sperm concentration over 20 × 10^6^. All cases of live delivery by natural conception occurred in patients with at least 40% motile sperm. In our study, TMSC strongly predicts spontaneous pregnancy after reconstruction in a group of primitive epididymal OA. A TMSC of 37.30 × 10^6^ has a sensitivity of 87.5% and a specificity of 85% to predict a spontaneous pregnancy occurrence after microsurgical reconstruction.

In couples with younger female partners, the baseline FSH level and TMSC after reconstruction could be good tools for counseling the couple to wait for a longer time to obtain natural conception after surgery. If a male with primitive epididymal OA with reconstruction and patency has a baseline low FSH level and/or a TMSC good enough for a natural pregnancy, it is advisable to wait for a natural pregnancy and not to proceed to ICSI.

Our study has some limitations. First, this was a single-center observational study on a small number of patients, so larger multicenter trials are needed to confirm our results. Second, it is a nonrandomized study.

## 5. Conclusions

Our data suggest that microsurgical VES is an effective therapy used in the treatment of men diagnosed with epididymal obstructive azoospermia. Lower FSH levels and higher TMSC at baseline were independent predictors for natural pregnancy occurrence after VES. As the sample size is not large enough, larger multicenter trials are needed to confirm our results.

## Figures and Tables

**Table 1:** Investigated parameters in study groups.

	All	With patency	With natural pregnancy	Without natural pregnancy	P*
Patients (number)	36	28	8	20	
Age (years)	34 [31–37]	33 [31–33]	30 [26–33]	37 [35–40]	0.145
Age of female partners (years)	32 [29–36]	31 [29–31]	30 [28.25–34.75]	31.5 [29–35.5]	0.411
Smokers (% yes)	38.9	39.3	62.5	30	0.199
Prior fertility status (% yes)	11.1	14.3	25	10	0.556
Medical history of urological infections (% yes)	44.4	53.6	37.5	60	0.381
FSH (UI/L)	3.86 ± 1.74	3.86 ± 1.66	2.84 ± 1.23	4.27 ± 1.66	0.023
LH (UI/L)	4.05 ± 1.58	4.1 ± 1.7	4.23 ± 1.69	4.00 ± 1.79	0.754
Testosterone (ng/mL)	5.70 ± 1.84	5.75 ± 1.9	5.47 ± 2.67	5.88 ± 1.67	0.701
Right testicular volume (cm^3^)	14.67 ± 5.38	14.57 ± 5.99	16.89 ± 3.97	13.59 ± 6.51	0.122
Left testicular volume (cm^3^)	13.92 ± 6.97	14.77 ± 7.03	16.73 ± 4.39	13.94 ± 6.84	0.251
Thickness of the right epididymis (mm)	8.90 ± 3.62	8.51 ± 3.40	8.75 ± 2.07	8.39 ± 3.95	0.775
Thickness of the left epididymis (mm)	8.68 ± 3.56	8.81 ± 3.27	9.66 ± 1.07	8.39 ± 3.90	0.238
TMSC (×10^6^)	0	50.14 ± 46.45	114.11 ± 37.65	24.55 ± 11.51	0.0001

TMSC: total motile sperm count, FSH: follicle stimulating hormone, and LH: luteinizing hormone.

*With natural pregnancy versus without natural pregnancy.

**Table 2:** Results of multivariate Cox proportional hazard model analysis.

Variable	Sig.(*P*)	Exp(*B*)	95.0% CI for Exp(*B*)
Age	0.163	1.543	0.839–2.838
Age of female partners	0.123	0.552	0.259–1.175
Medical history of urological infections (yes)	0.754	0.530	0.010–27.989
Prior fertility status (yes)	0.778	0.599	0.017–21.068
FSH	0.032	0.215	0.052–0.879
LH	0.114	4.019	0.715–22.581
Testosterone	0.465	0.799	0.438–1.459
TMSC	0.012	1.001	1.000–1.001

TMSC: total motile sperm count; FSH: follicle stimulating hormone; LH: luteinizing hormone.
